# The Spectroscopic Similarity between Breast Cancer Tissues and Lymph Nodes Obtained from Patients with and without Recurrence: A Preliminary Study

**DOI:** 10.3390/molecules25143295

**Published:** 2020-07-21

**Authors:** Joanna Depciuch, Agata Stanek-Widera, Nadia Khinevich, Hanna V. Bandarenka, Michal Kandler, Vadim Bayev, Julia Fedotova, Dariusz Lange, Jadwiga Stanek-Tarkowska, Jozef Cebulski

**Affiliations:** 1 Institute of Nuclear Physics Polish Academy of Sciences, PL-31342 Krakow, Poland; 2 Faculty of Medicine, University of Technology, Rolna 43, 40-555 Katowice, Poland; aswidera502@gmail.com (A.S.-W.); dlange693@gmail.com (D.L.); 3 Laboratory of Applied Plasmonics, Belarusian State University of Informatics and Radioelectronics, 220013 Minsk, Belarus; khinevichnadia@gmail.com (N.K.); h.bandarenka@bsuir.by (H.V.B.); 4 Polytechnic School, Arizona State University, Mesa, AZ 85212, USA; 5 Institute of Physics, University of Rzeszow, College of Natural Sciences, PL-35959 Rzeszow, Poland; michal.kandler.a@gmail.com (M.K.); cebulski@ur.edu.pl (J.C.); 6 Research Institute for Nuclear Problems of Belarusian State University, 220030 Minsk, Belarus; vadghen@gmail.com; 7 Institute of Agricultural Sciences, Land Management and Environmental Protection, University of Rzeszow, PL-35959 Rzeszow, Poland; jagodastanek@wp.pl

**Keywords:** breast cancer tissues, lymph nodes, recurrence, FTIR, Raman spectroscopy, chemical changes

## Abstract

Lymph nodes (LNs) play a very important role in the spread of cancer cells. Moreover, it was noticed that the morphology and chemical composition of the LNs change in the course of cancer development. Therefore, finding and monitoring similarities between these characteristics of the LNs and tumor tissues are essential to improve diagnostics and therapy of this dreadful disease. In the present study, we used Raman and Fourier transform infrared (FTIR) spectroscopies to compare the chemical composition of the breast cancer tissues and LNs collected from women without (I group-4 patients) and with (II group-4 patients) recurrence. It was shown that the similarity of the chemical composition of the breast tissues and LNs is typical for the II group of the patients. The average Raman spectrum of the breast cancer tissues from the I group was not characterized by vibrations in the 800–1000 cm^−1^ region originating from collagen and carbohydrates, which are typical for tumor-affected breast tissues. At the same time, this spectrum contains peaks at 1029 cm^−1^, corresponding to PO^2−^ from DNA, RNA and phospholipids, and 1520 cm^−1^, which have been observed in normal breast tissues before. It was shown that Raman bands of the average LN spectrum of the II group associated with proteins and carbohydrates are more intensive than those of the breast tissues spectrum. The intensity of the Raman spectra collected from the samples of the II group is almost three times higher compared to the I group. The vibrations of carbohydrates and amide III are much more intensive in the II group’s case. The Raman spectra of the breast cancer tissues and LNs of the II group’s samples do not contain bands (e.g., 1520 cm^−1^) found in the Raman spectra of the normal breast tissues elsewhere. FTIR spectra of the LNs of the I group’s women showed a lower level of vibrations corresponding to functional group building nucleic acid, collagen, carbohydrates, and proteins in comparison with the breast cancer tissues. Pearson’s correlation test showed positive and more significant interplay between the nature of the breast tissues and LN spectra obtained for the II group of patients than that in the I group’s spectra. Moreover, principal component analysis (PCA) showed that it is possible to distinguish Raman and FTIR spectra of the breast cancer tissues and LNs collected from women without recurrence of the disease.

## 1. Introduction

The lymph nodes (LNs) are very important functional parts of an immune system. They are responsible for supporting interactions between all cells in the immune system, especially B and T cells, stromal cells, and antigen-presenting cells [1]. They work like a channel for receiving and transmitting cells and immunogenic substances to the blood [2]. Moreover, the function, morphology, and structure of the LNs can be changed during pathological conditions in a body [3]. This is especially important in cancer metastasis because primary cancer cells spread to distant organs through the LNs [4,5]. Thus, the LNs can be used as markers of metastasis of aggressive cancer, including breast cancer [6,7,8]. However, molecular analysis techniques, which are conventionally applied to determine the features of the LNs’ characteristics for the good and bad prognosis in primary breast tumors, are still not efficient [9,10,11,12]. Moreover, histopathology staging of the LNs based on a study of the morphology features is also ineffective because it depends on laboratory staff, who perform microscopy analysis [13]. An additional difficulty for accurately investigating LNs is the great diversity of their structure [14]. Therefore, there is an urgent need for a more sensitive and objective method of LN analysis for breast cancer diagnostics [15].

Raman and infrared (IR) spectroscopies are methods that provide detailed information about chemical composition of different matter including biological samples. Using these techniques it is possible to determine vibrations of functional groups that build carbohydrate, deoxyribonucleic acid (DNA), protein and lipid compounds [16]. Moreover, these vibrational spectroscopies show even slight changes in molecules of a sample that occur upon influence of external factors, e.g., medical treatment [17,18,19].

Despite Raman and IR spectra being observed due to molecular vibrations, their physical fundamentals are different, resulting in mutual complementarity of these methods [20]. Therefore using both Raman and IR spectroscopies opens an opportunity to determine the complete chemical structure of analyzing samples.

Many papers have reported using vibrational spectroscopies to determine chemical changes in breast [21], lung [22], bone [23], thyroid [24], and brain [25] under carcinogenesis. Moreover, spectroscopy data are based on creating physical models, which can be used in monitoring cancer treatment processes. It has been shown that IR spectroscopy provides detection of changes between lymphoid tumors [18,19,20,21,22], as well as T and naive B lymphocytes [26]. Isabelle et al. managed to distinguish cancerous malignant and benign esophageal LNs by Raman and Fourier transform IR (FTIR) spectroscopies [15]. In addition, Liu et al. studied metastatic and non-metastatic LNs obtained from patients with thyroid cancers [27]. They showed that differences between these two types of LNs have the same amount and structure of IR regions corresponding to nucleic acids, proteins, lipids, and carbohydrates. Remarkably, they achieved rather high sensitivity and specificity of around 80.3% and 91.0%, respectively.

Considering the role of the LNs in a metastatic process and difficulties in diagnostics of these immune structures, an objective of this study was to determine and compare the chemical composition of the cancerous breast tissues and LNs of women who have recurrence, with those of women without recurrence. For this purpose, the results of Raman and FTIR spectroscopic measurements were combined with computational analysis. In more detail, we pursued a goal to show if there are any chemical similarities between breast cancer tissues and LNs, which can determine the efficiency of treatment.

## 2. Results and Discussion

**Figure 1:**
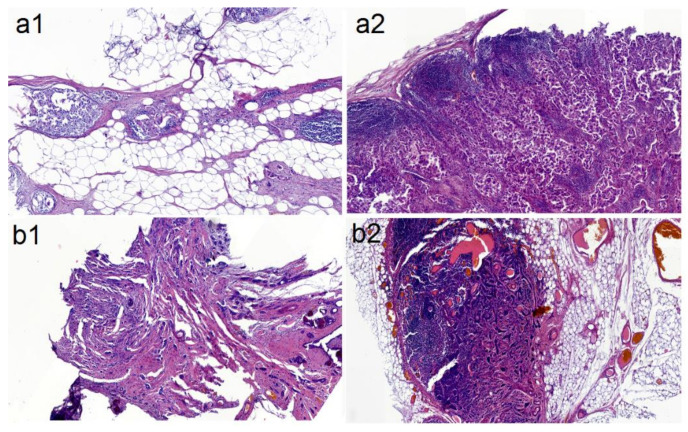
Microscopy images of the (**1**) breast cancer tissues and (**2**) LNs collected from the (**a**) I and (**b**) II groups of patients.

Figure 1 shows microscopy images of invasive breast carcinoma of no special type (1) and the metastasis of breast cancer to the LNs (2). The breast tissues and LNs were prepared so that the materials examined contained primary and metastatic cancer.

To determine differences in chemical composition between the breast cancer tissues and LNs collected from women with and without recurrence of the cancer, Raman and FTIR measurements were performed. In the obtained spectra variation of Raman bands’ intensities and FTIR bands’ maximum absorbance, as well as the shift of characteristic peaks and their presence/absence were noticed. Description of peaks’ characteristics for the Raman and FTIR spectra of the breast cancer tissues and LNs are presented in Table 1.

**Figure 2:**
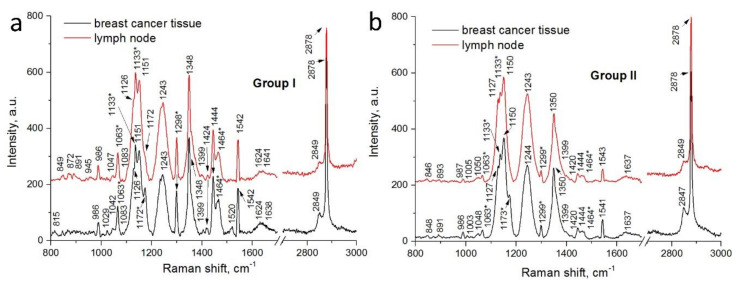
Average Raman spectra of the breast cancer tissues and LNs collected from patients of the (**a**) I and (**b**) II groups. The Raman bands typical for paraffin are marked with asterisks.

Figure 2 presents the Raman spectra registered for the breast cancer tissues and LNs of two patients’ groups. In general, all of the spectra have a view and contain the vibration bands typical for living tissues [28,29]. On the other hand, three bands at 1126–1151 cm^−1^, 1243–1244 cm^−1^ and 1348–1350 cm^−1^ are extremely prominent. The number of factors induces such high intensity of these bands. In particular, the first region is enhanced due to the contribution of paraffin (1133 cm^−1^) and carbohydrates (1150 cm^−1^), which are products of the malignant cells’ activity. The others (amide III and CH vibrations) are associated with changes in proteins observed upon tissues’ fixation [30]. In addition, the intensity of the proteins’ Raman bands depends on the angle of the incident light and in our case, on bonds’ orientation to the perpendicular laser beam. The so-called sinusoidal behavior of anisotropy (observed for the three above-mentioned Raman bands) is related to the global Raman tensor for the protein structure [31]. It should also be noted that the Raman spectra are characterized by a relatively weak amide I band (1637–1641 cm^−1^) associated with the alpha-helix protein structure. As a rule, this band is more intensive in the Raman spectra of cancer tissues [29]. However, the Raman analysis of the breast cancer tissues reported elsewhere [28] also showed a moderate amide I signature. What is more tissue infiltration with paraffin is known to inhibit several Raman bands of tissues including amide I [30].

Figure 2a shows the Raman spectra of the breast cancer tissues and LNs collected from the women without the recurrence of the cancer disease. The Raman signatures look rather identical but their careful comparison reveals some differences in patterns. Raman bands of the average LNs’ spectrum associated with the functional groups of nucleic acid, collagen, proteins and lipids are more intensive than those of the breast cancer tissues’ spectrum. Moreover, the latter one is not characterized by the vibrations in the range from 800 cm^−1^ to 1000 cm^−1^ originating from collagen and carbohydrates, typical for tumor-affected breast tissues [28]. This effect can take place due to higher density of the LNs but, what is more important, this can be caused by inhibition of tumor cells’ activity that results in a small amount of carbohydrates. At the same time, the Raman spectrum of the breast cancer tissue contains peaks at 1029 cm^−1^ (corresponding to PO^2−^ from DNA, RNA and phospholipids) and 1520 cm^−1^ (which was observed in normal breast tissues [28]).

Consequently, when the Raman spectra of the breast cancer tissues and LNs collected from women with recurrence of the cancer disease were compared (Figure 2b), we observed complete similarity of the obtained signatures. The Raman bands of the LN spectrums corresponding to proteins and carbohydrates are slightly more intensive than those of the breast tissues’ spectrum. At the same time, overall intensity of the Raman spectra collected from the samples of the II group of the patients is almost three times higher than that of the I group. In addition, the vibrations of carbohydrates and amide III are much more intensive in the case of the II group’s samples. The Raman spectra of both the breast cancer tissues and LNs do not contain even weak bands (e.g., 1520 cm^−1^) showing some affinity to the normal tissues as it was observed in the Raman spectrum of the breast cancer tissues taken from the patient without recurrence.

**Figure 3:**
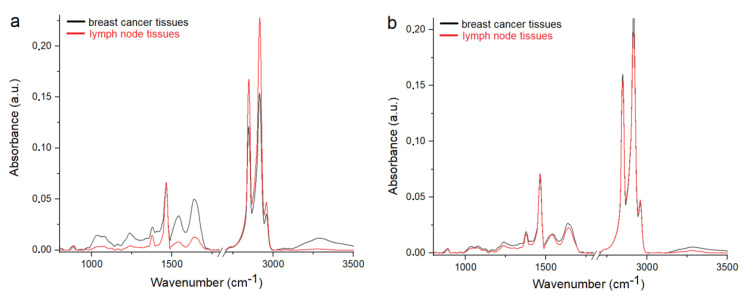
Average FTIR spectra of the breast cancer tissues and LNs from the (**a**) I and (**b**) II groups.

When we compared the FTIR spectra of the samples collected from the patients without (Figure 3a) and with (Figure 3b) recurrence, the similarity between the breast cancer tissues and LNs in the II group of patients can be noted. In the FTIR spectrum of the LNs from the I group, lower levels of DNA, RNA, phospholipids, carbohydrates and proteins were observed in comparison with the FTIR spectrum of the breast cancer tissues for the same group of patients. Moreover, significant shifts of peaks corresponding to nucleic acid, phospholipids (~930 cm^−1^) and lipids (~1740 cm^−1^) in the FTIR spectrum of the LNs were observed in comparison with the breast cancer tissues. Consequently, additional vibrations from the CH_3_ lipids group were visible in the LN spectra. Furthermore, very similar levels of each analyzed functional group building nucleic acids, carbohydrates, proteins and lipids can be noticed in the FTIR spectra of the breast cancer tissues and LNs collected from the women with recurrence of the cancer disease.

The description of peaks’ characteristics for the Raman and FTIR spectra of the breast cancer tissues and LNs are presented in Table 1.

Comparing the results obtained during the Raman and FTIR spectroscopies shows that spectra of the breast cancer tissues and LNs collected from women with recurrence of the disease are similar and do not completely coincide with the spectra collected from the samples of the no recurrence group. Therefore, it can be assumed that chemical changes in the breast tissues caused by cancer also induced changes in the LNs, which play a very important role in the spread of cancer disease [2]. Changes in the organism caused by pathological conditions, including cancer, have an influence on the morphology and chemical composition of the LNs. Lymphangiogenesis is the main change that occurs in the LNs during cancer [3]. Moreover, many human cancer cells seem to use the lymphatic system to promote the LNs’ metastases and the affection of distant organs [4,5,35]. The metastases of cancer cells very often take place through the LNs [36], where immunocompetent cells proliferate and, at the same time, produce inflammatory proteins such as cytokines [37]. The faster proliferation of cancer cells caused an increase of nucleic acid in the sample, which in the spectrum of the LNs collected from the women with recurrence of disease was observed as the same values of the Raman intensities corresponding to DNA and RNA functional groups in the spectrum of the breast cancer tissues (Figure 2b). Furthermore, in the samples collected from the women without recurrence of disease, a lower amount of glycogen and carbohydrate was noticed in comparison with the spectra of the breast cancer tissues, while in the spectra obtained from the II group of patients, the level of these chemical compounds is very similar in the breast cancer tissues and LNs. It is know, that an increase of glucose metabolism occurs in cancer cells. This growth causes a spike of oxygen consumption in metastatic LNs [38], which is observed in the spectra as a high level of glycogen and carbohydrate functional groups. Liu et al. reported similar results [27]. In the obtained FTIR spectra of the metastatic LNs, they noticed a higher level of carbohydrates in comparison with the non-metastatic tissues [27]. Moreover, they also found that the levels of proteins and lipids in the metastatic LNs are very similar to those observed in cancer tissues, which are also in good agreement with results obtained in our experiment. Additionally, Isabelle et al. studied the Raman spectra of cancer and non-cancer LNs. They noticed chemical changes between these two samples, especially in the Raman region originating from the protein and lipid regions [15]. These are the functional groups, whose changes are shown in Figure 2. Moreover, Ya-Qi et al. showed that the level of lipids and proteins in the FTIR spectra in the LNs with cancer cells was higher than in the FTIR spectra collected from the LN without cancer cells [39]. In addition, Silva et al. showed differences in the chemical compositions in the IR region corresponding to proteins and lipids [40], which correspond with results presented in this work (Figure 3). These authors showed changes in the amount of lipids and proteins, as well in the structure of described chemical compounds.

To determine the correlation between the LNs and breast cancer tissues, Pearson’s correlation test was performed.

**Figure 4:**
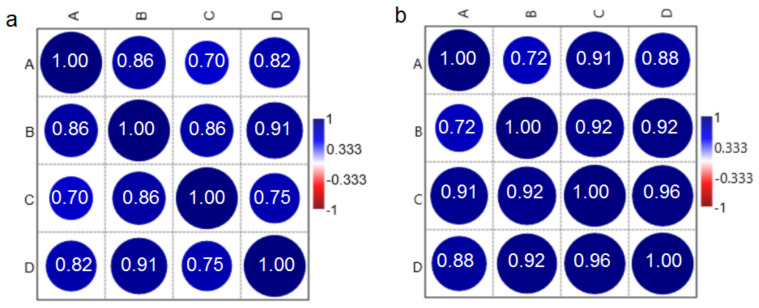
Pearson’s correlation test (N = 48) for the (A, C) breast cancer tissues and (B, D) LNs collected from women (A, B) without and (C, D) with the disease recurrence. Data were collected from the (**a**) Raman and (**b**) FTIR spectroscopies.

Pearson’s correlation test (Figure 4) was performed to investigate if it is possible to determine the correlation between the character/shape of the spectra obtained in this study. The data can be summarized as follows. A positive and significant correlation between the nature of the spectrum of breast tissue and LNs from the I and II groups of patients was noticed. However, the correlation obtained for the group of women without disease recurrence is less statistically significant than that for the II group. Pearson’s correlation test clearly showed that there is an interconnection between chemical changes occurring in the breast cancer and LN tissues and the possibility of metastasis or recurrence of the disease. Moreover, Pearson’s correlation test showed that the chemical compositions of breast cancer tissues are similar to that observed in the LNs. Interestingly, in Figure 4b, it was noticed that a more significant correlation between chemical compositions of the breast cancer tissues and LNs was visible for group of women with recurrence of the disease.

**Figure 5:**
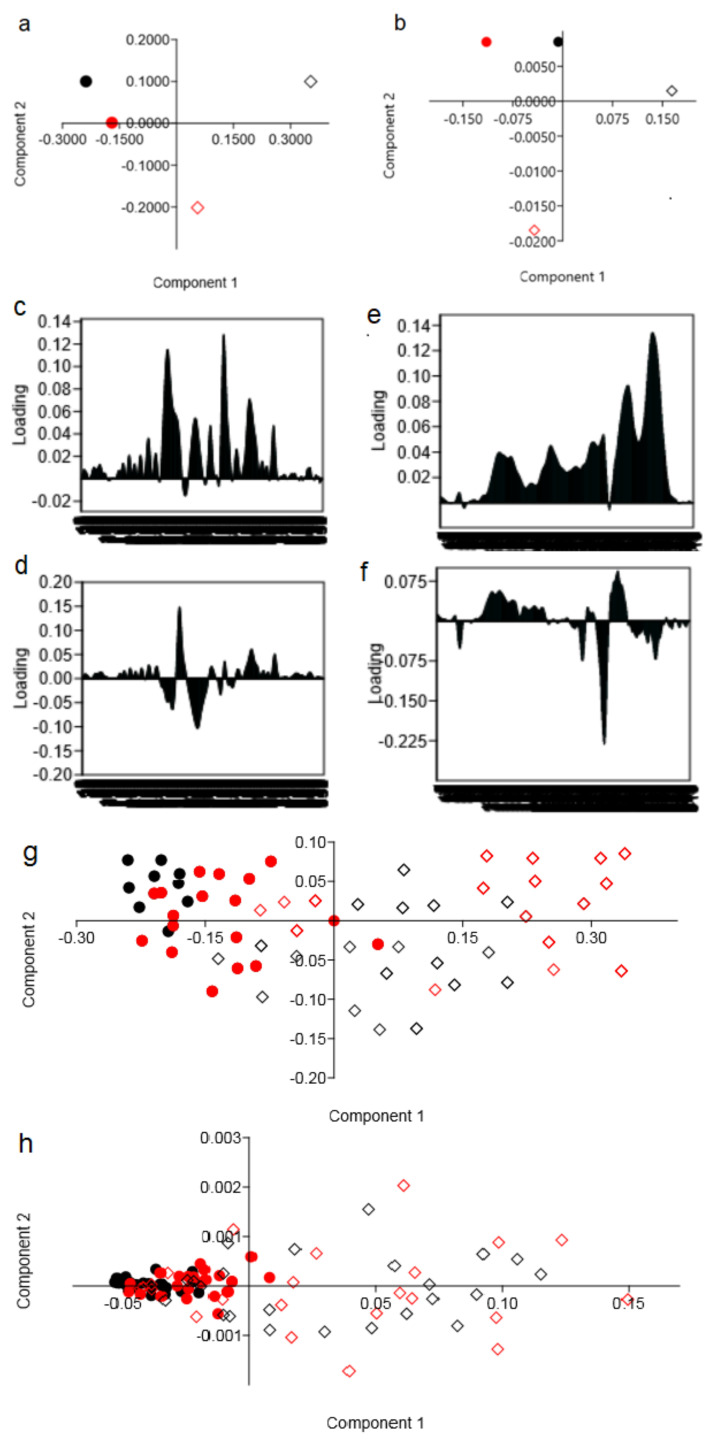
PCA of the breast tissues (black color) and LNs (red color) obtained from the (**a**,**g**) Raman and (**b**,**h**) FTIR spectra of the samples from the I (diamond) and II (dot) groups of patients, with loading plots of Components 1 and 2 of Raman (**c**,**d**) and FTIR (**e**,**f**). Due to differences in chemical compositions, a two-dimensional (2D) scores plot of the samples is presented through the selected spectral ranges between 800–1800 cm^−1^. Moreover, PCA analysis obtained for average spectra is presented in figures (**a**,**b**), and PCA analysis for all obtained spectra is presented in (**g**,**h**).

The significantly distinct morphological areas were spectroscopically discriminated using unsupervised exploratory PCA. Therefore, these areas can be analyzed separately to determine the significance of chemical differences between the breast cancer tissues and LNs collected from two analyzed groups of patients. The Raman (Figure 5a) and FTIR (Figure 5b) PCA plot showed separation of spectra obtained for breast tissues and LNs collected from the women without recurrence of cancer disease. While for the spectra of the same tissues, however, collected from the women with disease recurrence, PCA showed a lack of separation between spectra obtained for the breast cancer tissues and LNs. These results could suggest that Raman and FTIR spectra of the LNs collected from the women with disease recurrence were similar to those obtained for breast cancer tissues.

## 3. Materials and Methods

### 3.1. Materials

The study was conducted under the Institutional Review Board (Protocol No. KBET/6/06/2014) from June 2014 at the University of Rzeszow. The experimental protocols used in this study were approved by the institutional ethics committees (IECs) of the University of Rzeszow and were carried out in accordance with the approved guidelines. Consents were obtained from all the subjects.

Samples of the breast tissues and LNs were collected from eight female patients of the Maria Sklodowska-Curie Memorial Cancer Centre and the Institute of Oncology in Gliwice. All of the women were diagnosed with breast cancer but they showed different responses to treatment. Four patients had no recurrence after treatment and they formed the I analyzed group, while recurrence was observed for other women that formed the II group. The clinical characteristics of the patients are shown in Table 2.

#### Material Preparation

Materials collected during surgery were prepared in the same way as for the histopathological examinations. Thus, the tissues were placed in a liquid fixative for about 12 h. Next, ethanol within the tissue was gradually replaced with xylene, which varied in concentration from 50% to 99.8% with 10% increasing steps. Paraffin infiltration of the tissue was performed at a temperature of 52 °C, when infiltration sections were embedded in paraffin blocks, prepared by pouring liquefied paraffin into a metal mould and inserting thin piece of tissue with the appropriate spatial orientation. Pad paraffin-embedded breast and lymph nodes’ tissues were then cut into sections with a thickness of 10 μm using a microtome (Leica Biosystems) and next these slides were placed onto prepared slides made of CaF_2_ and inserted into the incubator at a temperature of 50 °C for 5 h to permanently adhere the sections to the surface of the slide. Prepared tissue samples and paraffin were measured separately. Then, the paraffin spectra were subtracted from the samples’ spectra. It was found that all characteristic Raman bands of paraffin did not prevent one from establishing the similarities/dissimilarities of the Raman spectra of the breast cancer tissues and LNs samples. Therefore, Figure 2 presents the Raman patterns of the samples before extraction of the paraffin spectrum, whose peaks are marked with asterisks. Moreover, for each obtained sample, an immunohistochemical diagnostics was done to confirm presence or absence of cancer cells.

### 3.2. Experimental

#### 3.2.1. Histological Images of Samples

Histological images of the measured tissue samples were taken using an Olympus BX 43 optical microscope (Tokyo, Japan) provided a 26.5 field diameter and equipped with Panoramic 250 Flash III slide scanner from 3D Histech. The magnification was 100×.

#### 3.2.2. FTIR Spectroscopy

During the FTIR measurements, a spectrometer (Bruker, Ettlingen, Germeny) Vertex 70v from Bruker was used with a diamond crystal ATR (attenuated total reflectance). Each sample was measured three times using 32 scans and a spectral resolution of about 2 cm^−1^. Then, an average spectrum for each sample was calculated and transformed from ATR to absorbance spectrum. Baseline correction, vector normalization and smoothing using nine points were applied for each obtained FTIR spectrum. The acquisition time was 30 s. All spectra were analyzed using OPUS software (Bruker Optik GmbH 2011, Ettlingen, Germeny).

#### 3.2.3. Raman Spectroscopy

We used the 3D scanning laser spectrometer Confotec NR500 (SOL Instruments Ltd., Minsk, Belarus) coupled to an upright Nikon Ni-U confocal microscope for the Raman spectra registration. The measurements were performed with diode-pumped 785 nm laser (Integrated Optics model SLM). The laser light was focused on the sample with a 100× objective (NA 0.95) in the ~1–1.5 µm spot. A Peltier-cooled CCD camera (Proscan Electronic Systems, Scheuring, Germany) was used for detection of the spectra collected in a backscattering geometry. We probed signals in the 800–1800 cm^−1^ and 2800–3000 cm^−1^ ranges of vibrational frequencies with a 1.8 cm^−1^ resolution. Rayleigh scattering was blocked using Semrock long-pass edge filters. The laser had a 7.2 mW power after passing through an optical system of the spectrometer and the objective. We collected three Raman spectra in different points of each sample. Excitation time was 3 s during the single Raman measurement. The measurements were conducted at room temperature.

In more detail, the spectra were recorded in three random spots of each sample and carefully compared to reveal if there are any strong inconsistencies in the Raman signatures. No significant spectra variations were observed for the samples’ sets, i.e., spectra of the breast cancer tissues and LNs were nearly similar in the range of specific patient groups regarding the presence/absence, position and intensity of the characteristic bands. To compensate for slight differences in the Raman spectra that appeared to be dependent on the position in the sample, they were smoothed by the Savitzky–Golay method using nine points of a window and the 2nd polynomial order. Then the spectra were averaged for each group of women, and subjected to user defined baseline subtraction, which avoided hiding small peaks. To find characteristic peaks, we selected lines in the Raman spectra whose intensity was three times higher than that of a noise. It should be noticed that we performed the Raman spectroscopy of the tissue infiltrated with paraffin, i.e., the Raman bands of the fixating material were observed in the Raman patterns of the samples but they negligibly contributed to the tissues spectra. We revealed the paraffin bands from its pure spectrum and also following data reported elsewhere [29]. The paraffin peaks were marked with asterisks on the final averaged spectra.

#### 3.2.4. Statistical and Computational Analysis

The average position of Raman and FTIR peaks are represented as means ±SEM (the standard error of the mean). To determine a correlation between chemical changes in the LNs and occurrence or absence of recurrence, the Pearson’s test with *p* < 0.05 were considered statistically significant with 95% confidence. Moreover, to obtain information about the spectra variation among the occurrence or absence of recurrence, principal component analysis (PCA) was performed for the FTIR and Raman range between 800 cm^−1^ and 1700 cm^−1^. For the statistical and computational analysis, Past 3.0. software was used.

## 4. Conclusions

The chemical composition of the cancerous breast tissue and LNs collected from the women with and without recurrence of cancer was determined in the present study. For this purpose, the Raman and FTIR spectroscopies were used.

The obtained Raman and FTIR spectra showed similarity in the shape of patterns and in the vibrational bands’ values of Raman intensities and FTIR maximum absorbance between the breast cancer tissues and LNs collected from women with recurrence of the disease. However, the spectra of the breast cancer tissues and LNs of the women without disease recurrence were characterized by non-complete pattern similarity. In particular, the LN samples showed a lower level of functional groups building nucleic acids, collagen, carbohydrates and proteins in comparison with breast cancer tissues. Raman bands of the average LN spectra of the females without recurrence associated with nucleic acid, collagen, protein and lipid functional groups were more intense than those of the breast cancer tissues’ spectrum. An important result is detection of the 1520 cm^-1^ band in the Raman spectrum of the breast cancer tissue of the no recurrence patients, which was previously observed for the normal breast tissue (i.e., not for tumorous tissue) [30]. Raman bands of the LN spectra corresponding to proteins and carbohydrates were found to be slightly more intense than those of the breast tissue spectra. In general, an intensity of the Raman spectra collected from the samples of the II group of the patients was shown to be almost three times higher than that of the I group. The vibrations of carbohydrates and amide III were characterized by much higher intensity in the II group samples.

Furthermore, Pearson’s correlation test showed positive and significant correlation between the nature of the spectra of the breast tissue and LNs from the I and II groups of patients. However, correlation obtained for the group of women without disease recurrence is less statistically significant than that for the II group. Moreover, PCA showed that it is not possible to distinguish the Raman and FTIR spectra of the breast cancer tissues and LNs collected from the women with recurrence of the disease.

The obtained results indicate that the chemical compositions in the LN and breast tissues are more similar in the case of patients with recurrence of the disease.

## Figures and Tables

**Table 1:** Wavenumbers of the Raman and FTIR peak positions with corresponding vibrations in analyzed samples, where BCI and LNI mean the breast cancer tissues and LNs obtained from the I group, while BCII and LNII mean the breast cancer tissues and LNs obtained from the II group [16,17,18,19,20,21,22,23,24,25,26,27,28,32,33,34].

**Raman Spectra Peaks (cm^−1^)**				
**BCI**	**LNI**	**BCII**	**LNII**	**Vibrations**
	849	848	846	CCH from collagen [16]
	872–892	891	893	CCH from collagen [16,17,18,19]
	945			C-C glycogen [32]
	986	986	987	C-C glycogen [32]
		1003	1005	C-C from aromatic ring of phenylalanine [16,32]
1029				PO^2−^ from DNA, RNA and phospholipids [16]
1042	1047	1048	1050	C-C glycogen [16]
1083	1083			PO^2−^ from DNA, RNA and phospholipids [16,28]
1126	1126	1127	1127	C-N from proteins, C-C from DNA [16]
1151	1151	1150	1150	C-O from carbohydrates [17,18,19]
1243	1243	1244	1243	Amide III [21]
1348	1348	1350	1350	CH from lipids and proteins [20]
1399	1399	1399	1399	CH from lipids and proteins [21]
1424	1424	1420	1420	CH_2_ from lipids [21]
1440	1440	1440	1440	CH_2_ from lipids, proteins [16,20]
1542	1542	1541	1543	Amide II [18,21]
1624	1624			Amino acids residues, proteins [33,34]
1638	1641	1637	1637	Amide I, proteins [33]
2849	2849	2847	2849	CH_2_ from lipids [17,20]
2878	2878	2878	2878	CH_3_ from lipids [20,21]
**FTIR Spectroscopy Peaks (cm^−1^)**				
**BSI**	**LNI**	**BSII**	**LNII**	**Vibrations**
931	922	931	926	PO_3_^−2^ group from DNA, RNA and phospholipids [22]
1078	1078	1072	1078	C-O group from glycogen [23]
1181		1181	1183	PO_3_^−2^ group from DNA, RNA and phospholipids [24]
1238	1238	1238	1238	Amide III [25]
1377	1377	1377	1377	CH_2_ group from protein and lipids [25]
1484	1484	1484	1488	CH_2_ group from cholesterol [25]
1541	1539	1543	1537	Amide II [26]
1639	1639	1637	1637	Amide I [26]
1741	1730	1732	1732	CO vibrations from lipids [27]
2849	2848	2848	2848	Symmetric stretching vibrations of CH_2_ [26]
2914	2915	2916	2919	Asymmetric stretching vibrations of CH_2_ [26]
2958	2958	2955	2959	CH_3_ asymmetric stretching [26]

**Table 2:** Description of the samples, where “R” means right breast, “L”—left, “nst”—invasive carcinoma, DCIS (ductal carcinoma in situ)—formation of intraductal carcinoma, “NG”—nuclear grading, “G”—grading, “IM”—mitotic index, i.e., an amount of abnormal mitosis in 10 large fields of view, “ER”—status of the estrogen receptor ER (evaluated on one plus, two pluses and three pluses), “PR”—status of the progesterone receptor PR (evaluated on one plus, two pluses and three pluses), “HER”—status of HER-2 receptor (evaluated on one plus, two pluses and three pluses), only three pluses is a positive reaction, in “ypTNM” “y” means treatment, “p”—pathological evaluation, “T”—tumour size, “N”—state of the LN, “M”—information about the spread.

No	A Year of Birth	Breast	Type of Cancer	DCIS	NG	G	IM	ER	PR	HER	Type of Surgery	Tumor Size before Treatment [cm]	ypTNM	Treatment	Answer	Recurrence
23--3	1960	L	NST	0	3	3	11	(+++)	(+++)	(+)	amputation	2.5	ypT1cN2a	CT, RT	partial small	Without evidence of failure
25--3	1962	R	NST	1	2	2	7	(++)	(+++)	(+)	amputation	2.3 × 1.9 × 1.9	ypT1cN1a	CT, RT	partial small	Without evidence of failure
21--3	1949	L	NST	0	2	2	5	(+)	(+++)	(+)	amputation	3.6	ypT2N1a	CT, RT	partial small	Without evidence of failure
10--3	1954	L	NST	0	3	3	43	(−)	(−)	(−)	amputation	4.0	ypT2N1a	CT, RT	partial	Without evidence of failure
5--3	1950	R	NST	1	3	3	15	(++) po CT	(+) po CT	(+) po Ct	amputation	1.1	ypT1aN1a	CT, RT	partial large	Spread to the brain, DCIS in the second breast
14--3	1970	L	NST	1	1	1	0	(−)	(+)	(+++)	amputation	3.0	ypT1cN1a	CT, RT	partial large	Spread to the lymph nodes
20--3	1952	L	NST	0	2	2	5	(−)	(−)	(+)	amputation	5.5	ypT2N3a	CT, RT	partial small	Spread to the lymph nodes and recurence
22--3	1937	L	NST	0	2	2	4	(+)	(+)	(−)	amputation	4.0	ypT2N3a	CT, RT	partial small	Endometrial cancer, spread to bone, lung, local recurrence
